# Cost analysis of medical device spare parts

**DOI:** 10.12669/pjms.342.14245

**Published:** 2018

**Authors:** Guven Bektemur, Nedim Muzoglu, Mehmet Ali Arici, Melike Kaya Karaaslan

**Affiliations:** 1 Guven Bektemur, MD Spec. Assistant Professor, Department of Health Management, University of Health Sciences, Istanbul, Turkey; 2 Nedim Muzoglu, M.Sc. Electronic Engineer, Istanbul Beyoglu Public Hospital Union, Clinic Engineering Department, Istanbul, Turkey; 3 Mehmet Ali Arici, Biomedical Engineer, Istanbul Beyoglu Public Hospital Union, Clinic Engineering Department, Istanbul, Turkey; 4 Melike Kaya Karaaslan, M.Sc. Physics Engineer, Istanbul Beyoglu Public Hospital Union, Clinic Engineering Department, Istanbul, Turkey

**Keywords:** KEY WORDS: Clinical Engineering, Cost, Medical Device, Spare Part

## Abstract

**Objective:**

To establish estimation method on budget management of medical device spare parts and to evaluate the cost of medical device spare parts in affiliated hospitals of Istanbul Public Hospital Unions (PHUs).

**Methods:**

While this evaluation was performed, the relationship between paid cost for spare parts according to technological development level of device groups and total inventory value was used. Spare part cost analysis was carried out by using the normalized weighted arithmetic average method. Cost analysis of medical equipment spare parts of Istanbul PHUs was performed by using the data retrieved from Ministry of Health Business Intelligence Decision Support System for spending of spare parts in 2015.

**Results:**

The medical device spare part groups were categorized based on technological development. Among 1 to 6 PHUs, the cost ratios were acquired for high, middle, low and simple technology group as 17.31 – 40.08%, 29.14 – 43.36%, 22.62 – 27.44% and 8.16 – 11.89%, respectively. The ratio between the spare part and total inventory costs for 1-6 PHUs were calculated as 1.66%, 2.87%, 3.03%, 3.31%, 2.57% and 4.69% respectively. Expected rates based on normalized weighted method were obtained as follows; 5.76%, 4.67%, 5.31%, 4.87%, 4.34% and 4.27%.

**Conclusion:**

The expenditure analysis and budget planning for medical device spare parts in PHU could be predicted more accurately by taking into consideration the expected rate calculated by the normal weight method. In additon, the importance of Clinical Engineering Service Units in management of medical devices has been determined.

## INTRODUCTION

In recent years, there has been dramatic developments in healthcare technology which crucially influenced the cost of technology investment and also sustainability costs. Keeping pace with the new technology plays the foremost role in investment increase.^1^,^2^ Therefore, operation costs of new technology need to be traceable and controllable. The health reform that has been taking place in Turkey since 2003 has made a great difference in the functioning and structuring of the public health facilities which constitute the biggest portion of the health service providers’ area throughout the country. With the establishment of the Public Hospitals Administration of Turkey and the construction of the Public Hospital Unions (PHU), health service delivery has become a more accessible, traceable and controllable structure.^3^ In the PHU, it is aimed to decrease the service costs while increasing the quality of service with efficiency oriented studies. For this purpose, efficiency report cards have been established by the institution and biomedical services have become one of the major indicators in these reports cards. Clinical Engineering Service Units (CESU) has been established to efficiently carry out maintenance-repair-calibration and planning processes of medical devices constituting a significant part of health investments. It is mainly aimed to provide sustainable healthcare services by managing the medical device technologies in the CESU of PHUs. However, in the near-future health management policies, CESU would be inevitably responsible for the financial management and cost planning’s of medical devices. Therefore, CESUs have a major task based on traceability and controllability of operation costs.^4^ In developing countries, it is important to follow the cost-effective and sustainable health policies by taking into account the needs, priorities, resources and capacities in terms of the development of health services.^5^

Finance has a vital impact on the performance of the health system.^6^ The cost of spare parts in the financial management of medical devices plays an important role in operating costs of new technology.^7^ While total maintenance costs of medical devices are calculated, labor and spare parts costs must be included as well.^8^ Selection of the optimal spare parts and consumables in the medical device sector, where many companies are present for various brands, is important in terms of operating cost of the medical device.^9^ When evaluating this group costs, firstly, it is necessary to define what constitutes the group. In the European Commission 1993 report, the medical device is defined in a comprehensive manner. In the same document, “accessory” is defined as “a piece(s) which is not regarded as a medical device in its own right, but which is manufactured for use with this device in order to ensure proper use of the medical device”.^10^ Spare parts are defined as internal and / or external equipment which is not regarded as a stand-alone medical device but is compatible with the existing components and is supplied to replace the existing components of a device.^11^

**Fig.1:**
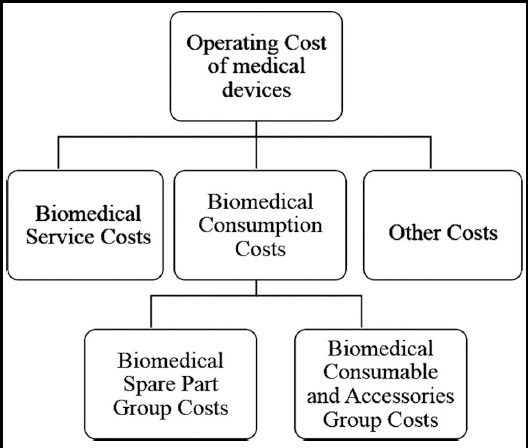
Classification for operating cost of medical devices.

Operating costs of medical devices can be classified as biomedical device associated consumption costs, service and maintenance costs and other costs. Afterwards, the costs of biomedical consumption is divided into biomedical spare parts and accessories (BSPA) as well as biomedical consumable group costs which includes main board, chassis parts, monitor parts, fundamental additional part (probs, electrodes, leads) and gels, solutions, filters, accessories respectively (Fig.1).

When the medical device operating costs are evaluated; medical device groups are classified as high, medium, low and simple technology which are based on technological development level and price values.^12^ When the expenditure of medical device spare parts are examined, it is revealed that there is a correlation between the acquisition prices and the maintenance costs of medical devices.^13^,^14^ The sale price of a medical device is one of the determinants regarding the medical device operational costs.^15^ There is also a direct proportionally relationship among accessories, spare parts, consumables and total medical device inventory.^7^ Similarly, Temple-Bird (2005) stated that, the cost expenses of previous years are examined; the ratio of the annual cost of spare parts to the total cost of acquisition is calculated approximately as 10% for high technology devices, 5% for medium technology, 1.25% for low technology and 0.25% for simple technology.^12^ Hence, medical device spare parts and expenditure can be used for future inventory planning and estimation on budget management.^7^

In our study, we first classified the biomedical spare part groups and explained the role of the CESU in the procurement process of these materials. Furthermore, according to the data of 2015, the cost of spare parts of all medical devices in Istanbul PHUs was taken and these costs were analyzed. In the cost analyses, it was aimed to establish an estimation method for the health managers and the CESU in the requirement planning. In the literature, approaches on the ratios of medical device operating costs to total medical device acquisition costs were evaluated in terms of annual biomedical spare parts purchasing costs in Istanbul PHUs.

## METHODS

**Fig.2:**
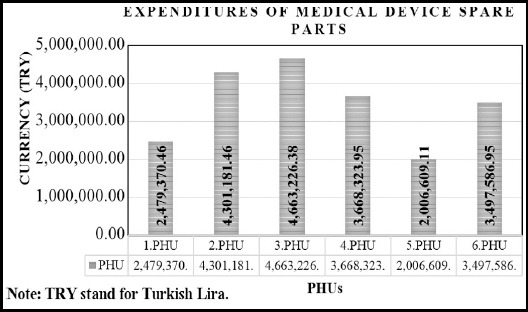
Expenditures of Medical Device Spare Parts for 2015.

Istanbul province is a valuable source for calculation of cost based on the healthcare technology due to both; the population which the health services are provided and the diversity of hospital service groups. Considering the geographical locations and access to the health facilities, Istanbul is divided into six PHUs. Each of them has 7-15 hospitals. The data required for the evaluation of spare part costs were obtained from the Ministry of Health Business Intelligence Decision Support System. PHUs are numbered from one to six, to protect the anonymity. Expenditures made for the spare parts group, except medical devices which has warranty throughout 2015, are shown in Fig.2.

Differences in spending on public hospital unions indicate that effective management of spare parts spending in health facilities should have a criterion. In order to determine the criterion for the ratio of medical device spare parts to the total acquisition cost of medical device inventory, it is necessary to divide the medical device inventory into technological development levels in terms of device groups and calculate the cost of each group separately. Primarily, to categorize devices according to the state of technological development, a technological development level classification has been defined for each biomedical product type. While these groups are identified, technical service costs and continuous service-maintenance requirements have been taken into consideration. By evaluating the acquisition costs and service-maintenance requirements of medical devices, we can list groups of certain devices as follows; first, high technology group; Radiotherapy, Diagnostic Radiology Devices etc., second, medium technology group; Anesthesia, Dialysis Systems, etc. These technology groups need continuous maintenance and certain parts replacement by only specialized technical staff. Third, the low-tech group; physiological signal monitoring and measurement systems such as ECG, Patient monitors, etc., finally, the simple technology group, nebulizers, aspirators, oxygen therapy devices, etc.

After such a classification process, the medical device inventory is analyzed, the numerical ratios and cost ratios of high, medium, low and simple technology groups are calculated. This calculation was conducted based on technological development of approximately 110.000 medical devices. Second, for each technology group the ratio between annual cost of spare part and total acquisition cost should be estimated. This same estimation method was followed as in the study of Temple-Bird.^12^ In order to calculate the ratio between the total expenditures of medical device spare parts and the total acquisition cost of medical devices, considering the obtained acquisition costs of technological development level and the spare parts expenditure ratios according to the technological development level which was taken as a reference, the normalized weighted arithmetic average method is applied.

To use this methodology, a data set and also weight functions for each unit of data set are required. Besides the sum of the weight functions are normalized to “1”. They are denoted by “w” for each data set in Equation 1.

In Equation 2, the normalized weighted arithmetic average value is defined as follows; the normalized weighted arithmetic average value “ŷ”, each unit of data set as “y” and the weight function for each unit data set as “w”.


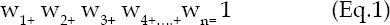



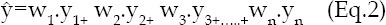


Based on the technology development level the ratio between the annual costs of the spare parts and the total acquisition cost for high, medium, low and simple level groups were taken 10%, 5%, 1.25%, 0.25% respectively as (yn).^12^

The weight functions for each unit of data set which are obtained by the technological development level classification analysis of medical device inventories are defined as the cost ratios of the total inventory according to technological class and they are shown as (wn). Consequently, a criterion is developed to assess the ratio of total medical device spare parts cost to total medical device acquisition cost.

## RESULTS

Medical device inventories belonging to Istanbul PHUs were classified according to the technology levels mentioned in the introduction, as high, medium, low and simple The numerical and cost ratios of technology groups within the total inventory are calculated and illustrated in Table-I.

**Table-I:** Technological Development Percentages of Medical Device Inventory of Istanbul PHUs.

PHU Name	High Technology Group		Middle Technology Group		Low Technology Group		Simple Technology Group	

	Numerical Ratio	Cost Ratio	Numerical Ratio	Cost Ratio	Numerical Ratio	Cost Ratio	Numerical Ratio	Cost Ratio
1.PHU	1.73%	40.08%	14.26%	29.14%	28.36%	22.62%	55.65%	8.16%
2.PHU	1.51%	24.03%	13.48%	38.07%	28.72%	26.93%	56.29%	10.97%
3.PHU	1.60%	33.48%	11.55%	32.80%	32.36%	24.29%	54.49%	9.43%
4.PHU	1.39%	26.32%	15.24%	37.97%	30.23%	25.59%	53.14%	10.12%
5.PHU	1.06%	20.54%	11.54%	37.60%	36.04%	30.97%	51.36%	10.89%
6.PHU	1.46%	17.31%	15.00%	43.36%	31.40%	27.44%	52.14%	11.89%

In order to make an accurate analysis of the spare parts costs of 2015, the medical device inventories within the warranty period throughout 2015 were not included in the calculation since we do not expect any spare parts, maintenance and repair expenditure for these devices, except malfunctions and damage caused by user error.

The ratio of spare parts expenditures for each PHU’s according to their inventory cost and normalized weighted arithmetic averages (ŷ) are calculated and shown in Table-II.

**Table-II:** Medical Device Spare Part Costs, Expenditure Ratios and “ŷ” values for 2015.

Public Hospital Unions	Total Acquisition Cost of Medical Devices (TRY)	Cost of Medical Device Spare Parts for 2015 (TRY)	Spare Part Costs / Inventory Rate for 2015	Expected Rate Calculated by Normalize Weight Method for 2015 (ŷ)
1.PHU	149.135.483	2.479.370	1.66%	5.76%
2.PHU	149.725.239	4.301.181	2.87%	4.67%
3.PHU	153.937.925	4.663.226	3.03%	5.31%
4.PHU	110.713.223	3.668.324	3.31%	4.87%
5.PHU	78.033.752	2.006.609	2.57%	4.34%
6.PHU	74.503.099	3.497.587	4.69%	4.27%

Examination of spare part costs in Istanbul province indicate that spare parts expenditure for each PHU’s with respect to their own inventory cost are between 1.66% and 4.69%. The underlying reasons for the differences in spare parts costs among the PHUs may be listed as management process differences in CESU, spare parts supply chain process differences and differences in the acquisition cost of medical device inventory, etc. Additionally, normalized weighted arithmetic averages (ŷ) are calculated to be between 4.27% and 5.76%.

## DISCUSSION

The technological development percentages of medical devices vary among PHUs as shown in Table-I. The numerical ratios of high technology group in all PHUs range from 1.06% to 1.73% whereas inventory cost from 17.31% to 40.08%. The underlying reason may be the direct proportion between the number of training / research hospitals and high technology devices in PHUs. Further, the PHUs which include predominantly oncological diagnosis and treatment centers, expectedly have higher ratios as they belong to high technolog group of inventory. On the other hand the low level technology group ratios are approximately equal.

The analysis regarding spare part expenditures of PHUs in Istanbul indicates that the total inventory costs differ between 1.66% to 4.69%. The differences may be due to variations in operation among Clinical Engineering Service Unions, the time distinctness of spare part supply chain and the procurement cost of medical device inventory etc. When Table-II is examined, almost all the PHUs in Istanbul are proportionately below the calculated “ŷ”; health facilities provide replacement parts with maintenance-repair agreements, especially in high-tech equipment which is one of the reason of low level ratios. For example in the 1st PHU, the total acquisition cost of devices that have undergone maintenance and repair contracts which includes also spare parts, is 28.315.047,20 TRY. This amount is 18.98% of total inventory cost. When we subtract this amount from the cost of total inventory, the new value of total inventory cost is calculated as. 120.820.435,80 TRY. According to the new value, the cost of spare parts and the normalized value are calculated as 2.05 and 5.00% respectively, in contrast with the values according to the total inventory cost which were calculated as 1.66% and 5.76%. Except one PHU; data about the devices covered with repair and maintenance agreements could not be retrieved. Even with these results, the total inventory cost is considerably lower than the expected normalized values. One reason may be that spare part supply costs are influenced by international price differentials in, as in the case of medicine, however further studies need to be carried out in order to validate this interpretation. Moreover, another reason may be the price variability, depending on whether the spare parts used in medical devices are original or not, but to support this view more evidence is necessary. Furthermore, it is important that the spare part costs are electronically coded in a proper way thereupon more accurate data analysis can be done. Additionally, spare part costs in the “parts-included” repair and maintenance agreements can be shown separately to improve the precision of the cost between maintenance and spare part costs. With these improvements, the data set for calculations should be more accurate. Thus, (ŷ) calculated by the normal weight method would be more meaningful criterion.

## CONCLUSION

It has been determined that spare parts should be categorized correctly in order to evaluate the spare parts costs realistically. In the procurement process for each different spare parts group, “Clinical Engineering Service Units” have a crucial role and spare parts costs, that are one of the key items in health technology expenditures, can be shown to be a predictable value. In order to calculate the biomedical spare part expenditure of all healthcare facilities, a criterion (ŷ) can be obtained by using the normal weighted arithmetic average method, taking advantage of the acquisition cost of medical equipment inventory and cost of spare parts according to technological development level. The expenditure analysis and budget planning for medical device spare parts in PHU could be predicted more accurately by taking into consideration the expected rate (ŷ) calculated by the normal weight method. In health facilities, CESU has a vital importance in the effective and efficient use of medical device technologies. The active role of the CESU in the spare parts procurement process will contribute to reducing the cost of spare parts and ensuring service sustainability. Healthcare managers who are looking for ways to overcome financial problems in healthcare spending could restructure the CESU in all areas where healthcare technology is used. Moreover, by the proposed restructuring, significant profitability in operating expenses can be more effectively realized.
